# The physiological and neuroendocrine correlates of hunger in the Red Junglefowl (*Gallus gallus*)

**DOI:** 10.1038/s41598-017-17922-w

**Published:** 2017-12-21

**Authors:** J. J. Lees, C. Lindholm, P. Batakis, M. Busscher, J. Altimiras

**Affiliations:** 1 IFM, University of Linköping, Linköping, Sweden; 2 Department of Animal Sciences, Wageningen University, Wageningen, Netherlands

## Abstract

The ability to regulate food intake is critical to survival. The hypothalamus is central to this regulation, integrating peripheral signals of energy availability. Although our understanding of hunger in rodents is advanced, an equivalent understanding in birds is lacking. In particular, the relationship between peripheral energy indices and hypothalamic ‘hunger’ peptides, agouti-related protein (AgRP), pro-opiomelanocortin (POMC) and neuropeptide Y (NPY) is poorly understood. Here, we compare AgRP, POMC and NPY RNA levels in the hypothalamus of Red Junglefowl chicks raised under *ad libitum*, chronic restriction and intermittent feeding regimens. Hypothalamic gene expression differed between chronically and intermittently restricted birds, confirming that different restriction regimens elicit different patterns of hunger. By assessing the relationship between hypothalamic gene expression and carcass traits, we show for the first time in birds that AgRP and POMC are responsive to fat-related measures and therefore represent long-term energy status. Chronically restricted birds, having lower indices of fat, show elevated hunger according to AgRP and POMC. NPY was elevated in intermittently fasted birds during fasting, suggesting a role as a short-term index of hunger. The different physiological and neuroendocrine responses to quantitative versus temporal feed restriction provide novel insights into the divergent roles of avian hunger neuropeptides.

## Introduction

The ability to balance energy acquisition and expenditure is fundamental to life. The networks controlling hunger and satiety must ensure that the metabolic needs of an individual are met over multiple temporal scales. Plasticity in feed intake regulation may be particularly relevant to avian species, which commonly experience periods of food restriction, for example during migration, incubation, overwintering and overnight^1^. Despite this plasticity, our mechanistic understanding of avian hunger is poor in comparison to that of rodent hunger, which has been extensively studied. Much of the available data stem from studies involving domesticated breeds, in particular broiler chickens, which have been selected for increased feed intake and growth and are therefore not representative of wild birds^2^. An increased, ecologically relevant understanding of avian hunger therefore requires equivalent data from non-domesticated species.

Within the basal hypothalamus, the arcuate nucleus is central to energy homeostasis and the regulation of hunger, integrating peripheral metabolic signals of energy status into an appropriate appetitive response. Circulating hormones and metabolites cross the blood-brain barrier where they are detected by specific neuronal populations within the arcuate nucleus^3^. This peripheral metabolic information is then integrated with limbic, sensory and circadian inputs to form an overall perception of hunger and appetite. Although much of our mechanistic knowledge regarding the hypothalamic control of hunger stems from mammalian research, the primary cell populations involved are conserved between mammals and birds^4^. In particular, the orexigenic neuronal populations in the arcuate nucleus stimulating food intake are those co-expressing the neuropeptides, neuropeptide Y (NPY) and agouti-related peptide (AgRP). Conversely, anorexigenic neuronal populations inhibiting food intake are those co-expressing pro-opiomelanocortin (POMC) and cocaine- and amphetamine-regulated transcript (CART)^3^.

Current knowledge regarding the appetitive roles of AgRP, NPY and POMC in the arcuate nucleus of birds stems mainly from feed restriction experiments. Individuals are either chronically feed restricted or exposed to an acute period of restriction after which hypothalamic gene expression is quantified. AgRP is an antagonist of the anorexigenic MC4R receptor and therefore stimulates food intake. Chronic, quantitative feed restriction elevates AgRP expression in the basal hypothalamus of broiler breeder hens^5^, consistent with findings in mammals^6^. Upon 2 days of *ad libitum* refeeding, however, AgRP expression levels decrease by around 75%, indicating a rapid hypothalamic AgRP response to the level of feed. This rapid response is further illustrated by experiments involving acute, 24 hour food restrictions, which also elevate AgRP expression within the hypothalamus of broiler chicks^7^ and in the arcuate nucleus of young Japanese quail^8^. It has been proposed that, as in mammals, this AgRP response is mediated by a depletion of peripheral energy stores, such as adipose tissue although a direct link between stored energy and AgRP expression is yet to be demonstrated explicitly^9^. The pancreatic polypeptide NPY has been shown to stimulate food intake in diverse avian taxa and operates via the NPY Y_1_ and Y_5_ receptors^10^. Its hypothalamic expression pattern upon chronic and acute feed restriction is therefore often parallel to that of AgRP, albeit at an apparently reduced magnitude^11,12^.

The melanocortin precursor, POMC acts antagonistically to AgRP via its cleavage product, α-melanocyte-stimulating hormone, which inhibits food intake after central injection^13,14^. Although POMC expression is associated with decreased food intake and increased energy expenditure, the response of POMC to feed restriction is less consistent than that of AgRP and NPY in birds. For example, neither chronic nor acute restriction elicit measurable changes in POMC in the arcuate nucleus of broiler breeder hens^5^ or Japanese quail^8^, respectively. However, studies in broiler chicks show both reduced^7,15,16^ and unchanging^12^ levels of POMC upon feed restriction. Together, these observations may indicate that the orexigenic effects of AgRP and NPY expression are more important in driving a hunger response to feed deprivation than the inhibition through POMC^17^. Alternatively, the posttranslational control of POMC may underlie its physiological role, meaning that mRNA levels alone are not indicative of functionality.

Although the central neuronal populations driving hunger are largely conserved between mammals and birds, the peripheral signals to which they respond differ. The adipose-derived peptide, leptin elicits a reduction in feeding intensity in mammals through its upregulation of *POMC* and downregulation of *AgRP* ^18^. Although peripheral and central administration of leptin reduces feeding many avian species, low expression of the leptin gene in adipose tissue and low circulating levels of the peptide suggest a reduced role for energy balance regulation in birds^19^. Similarly, ghrelin, a gut-derived peptide, induces feeding in mammals but appears to reduce feed intake in birds^20^, although the existing data may be biased by a predominance of studies in poultry. As appetite has been heavily selected upon in many of these domesticated breeds, their representativeness as a model for avian hunger is questionable. Given the apparent divergence of function in evolutionarily conserved ‘hunger’ peptides between birds and mammals, comparative studies are likely to yield novel insights into the plasticity and evolution of energy homeostasis in vertebrates.

Given the strong association required between external indicators of energy availability, their circulating representative signals and subsequent expression of hypothalamic ‘hunger genes’, physiological indicators of energy status would be expected to show a correlation with hypothalamic gene expression. Determining such correlations doesn’t require a knowledge of the intermediate representative signals but would provide a basis for comparing the avian and mammalian control of appetite. In particular, the level of adipose tissue (i.e. stored energy) should show a strong relationship with the hypothalamic expression of AgRP and POMC if the neural circuitry controlling hunger is conserved between birds and mammals. Similarly, we might expect the level of gizzard and crop fill to correlate with hypothalamic gene expression if satiety is influenced by signals relevant to the level of ingested food. However, few studies have simultaneously measured physiological indices of energy availability and hypothalamic gene expression. Here, we quantify carcass traits and relative mRNA expression levels within the basal hypothalamus of Red Junglefowl raised under differing feeding regimens to test the hypothesis that physiological indices of energy status are reflected in the expression levels of appetitive genes within the hypothalamus. As the wild ancestor of domestic chicken breeds, the Red Junglefowl is an ideal species in which to both investigate the neuroendocrine basis of avian hunger as well as to contextualise existing data for farmed birds and assess the impacts of domestication upon hunger/satiety networks.

## Results

### Body mass and food intake

**Figure 1:**
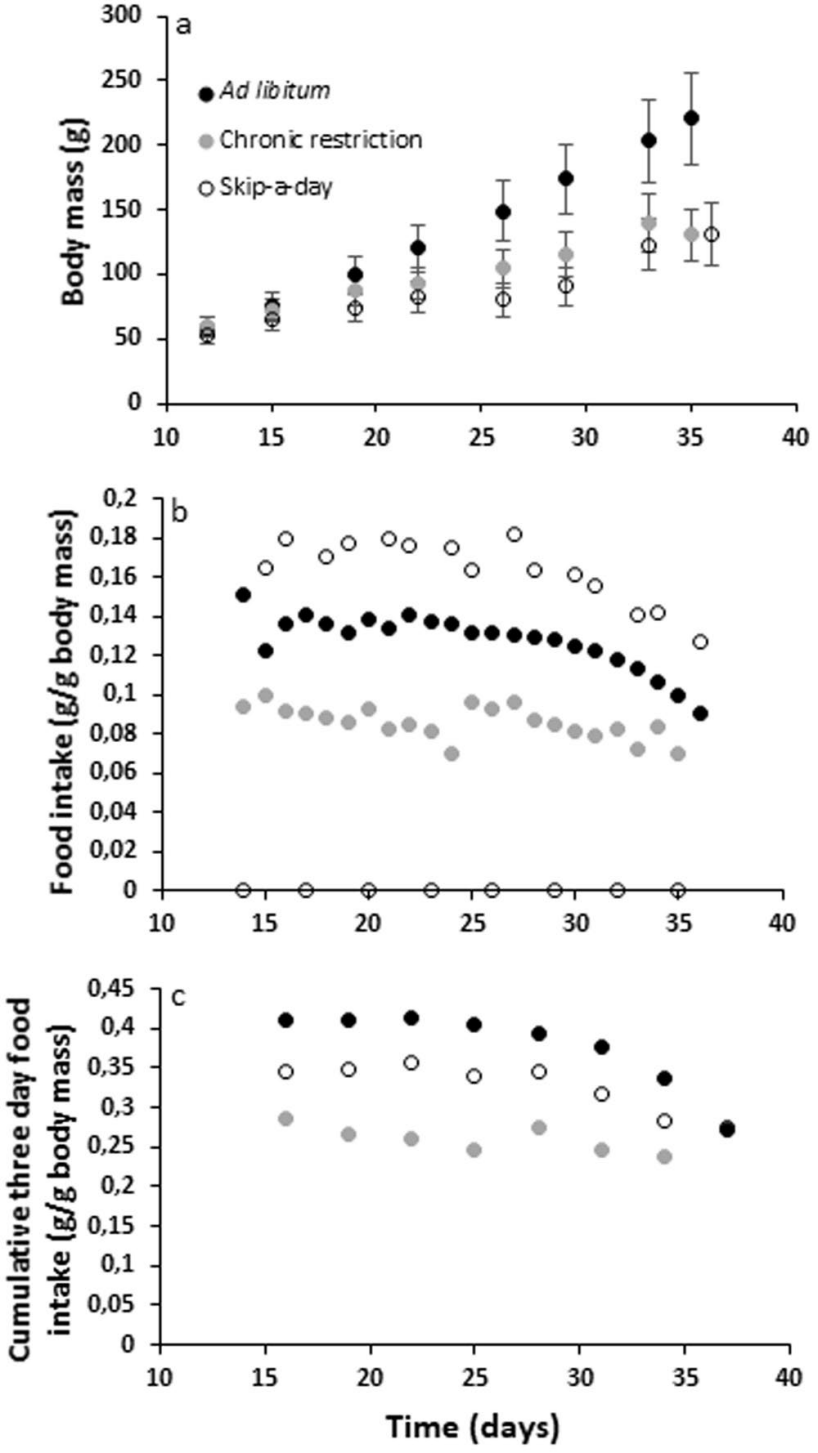
Body mass trajectories (**a**), food intake (**b**) and cumulative food intake (**c**) of Red Junglefowl chicks reared under *ad libitum* feeding (AL, black circles), chronic feed restriction (CR, grey circles) and skip-a-day feeding (S, open circles). Final body masses were higher in AL birds (N = 48) in comparison to CR (N = 48) and S birds (N = 43) as determined using two-way ANOVA. Error bars represent standard deviations of the mean.

Mean body mass at day 35–36 was higher in *ad libitum* (AL) birds (220.6 ± 5.1 g) in comparison to chronically restricted (CR) birds (130.6 ± 2.9) and intermittently fed skip-a-day birds (131.7 ± 3.7) (Fig. 1a). Sex differences in body mass were only apparent in AL birds in which males were, on average, heavier than females. Food intake (g/g_body weight_) was lower in CR birds as a result of their 70% feed restriction (Fig. 1b). On fed days, however, birds on a skip-a-day regimen ate more than those in the AL group. Despite their higher intake on fed days, birds on a skip-a-day regimen were unable to achieve the equivalent cumulative food intake of AL birds over a 3-day skip cycle (Fig. 1c). Cumulative 3-day food intake in skip-a-day birds was, however higher than that of CR individuals.

### Hypothalamic gene expression

**Figure 2:**
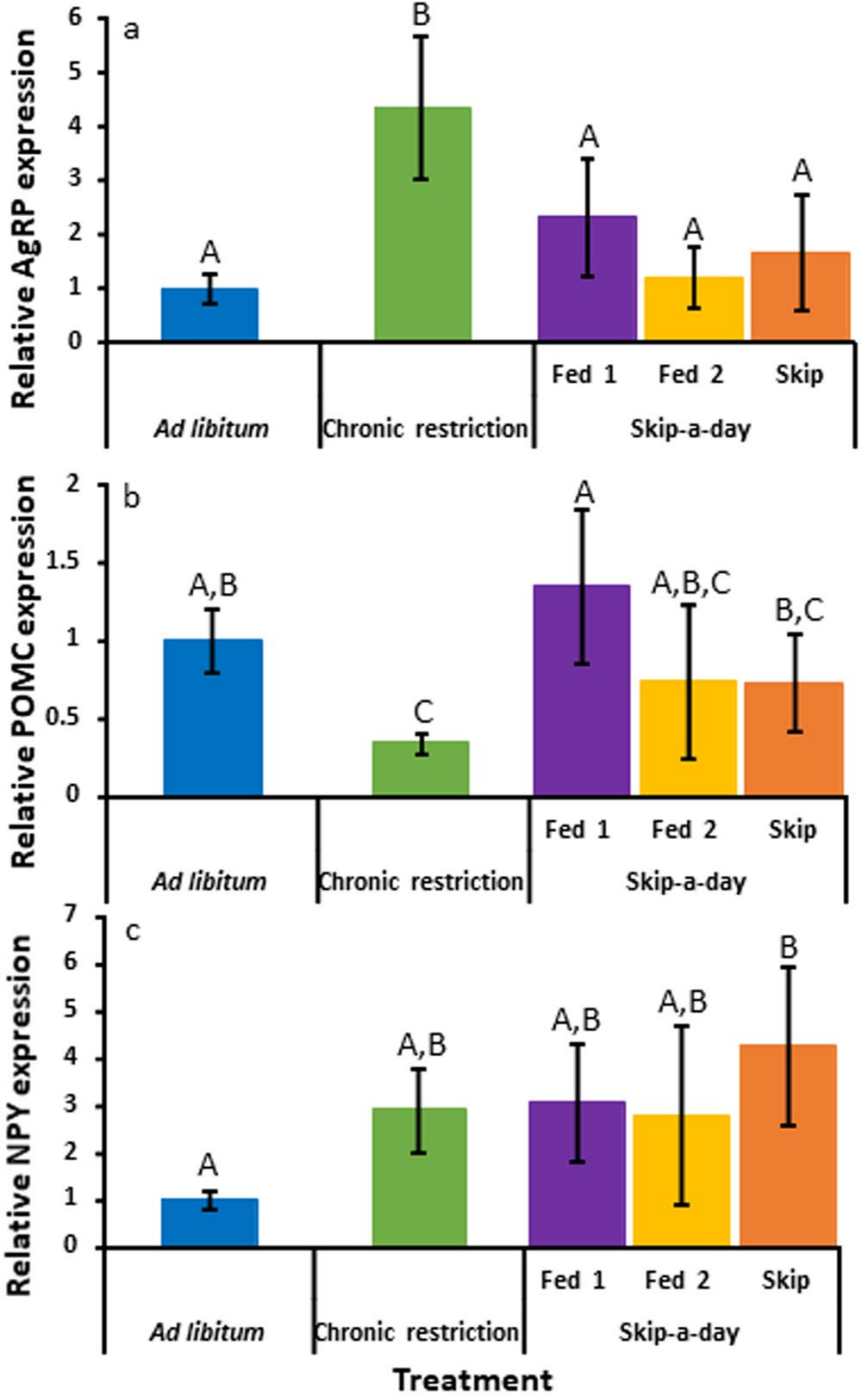
Gene expression of agouti-related peptide (AgRP) (**a**), pro-opiomelanocortin (POMC) (**b**) and neuropeptide Y (NPY) (**c**) in the arcuate nuclei of Red Junglefowl chicks reared under *ad libitum* feeding (blue, N = 6), chronic feed restriction (Green, N = 6) and skip-a-day feeding consisting of a first fed day, Fed 1 (purple, N = 5) a second fed day, Fed 2 (yellow, N = 4) and a skipped day without food (orange, N = 7). Expression levels represent mRNA levels relative to those of the housekeeping gene, GAPDH. Error bars represent standard deviations of the mean. Significant differences (determined using two-way ANOVA, p < 0.05) are indicated with different letters.

AgRP expression in the basal hypothalamus differed between CR birds and all other feeding treatments (Fig. 2a: ANOVA: Treatment, F_4,24_ = 11.3, r^2^ = 0.6, p < 0.001). Specifically, AgRP expression in CR birds was 4.4 times that of AL birds. AgRP expression was, however, unaffected by the skip-a-day feeding regimen and was unchanged on the first day of feeding (Fed1), the second day of feeding (Fed2) and the fasting day (S) in skip-a-day birds. POMC expression differed between treatment groups (ANOVA: Treatment, F_4,24_ = 7, r^2^ = 0.5, p < 0.01) and in CR birds showed an expression pattern opposite to that of AgRP, decreasing to 0.34 that of AL controls (Fig. 2b). Although expression in Fed1, Fed2 and S birds was not different from AL controls, within the skip-a-day regimen POMC levels were higher in Fed1 birds compared to both S and CR birds. NPY expression was elevated in CR, Fed1, Fed2 and S birds compared to AL individuals (Fig. 2c, ANOVA: Treatment, F_4,24_ = 5.3, r^2^ = 0.5, p < 0.01). This difference was, however, only significant between AL and S birds. The intermediate NPY values of CR, Fed1 and Fed2 birds were not different from those of AL or S individuals.

### Carcass traits

**Figure 3:**
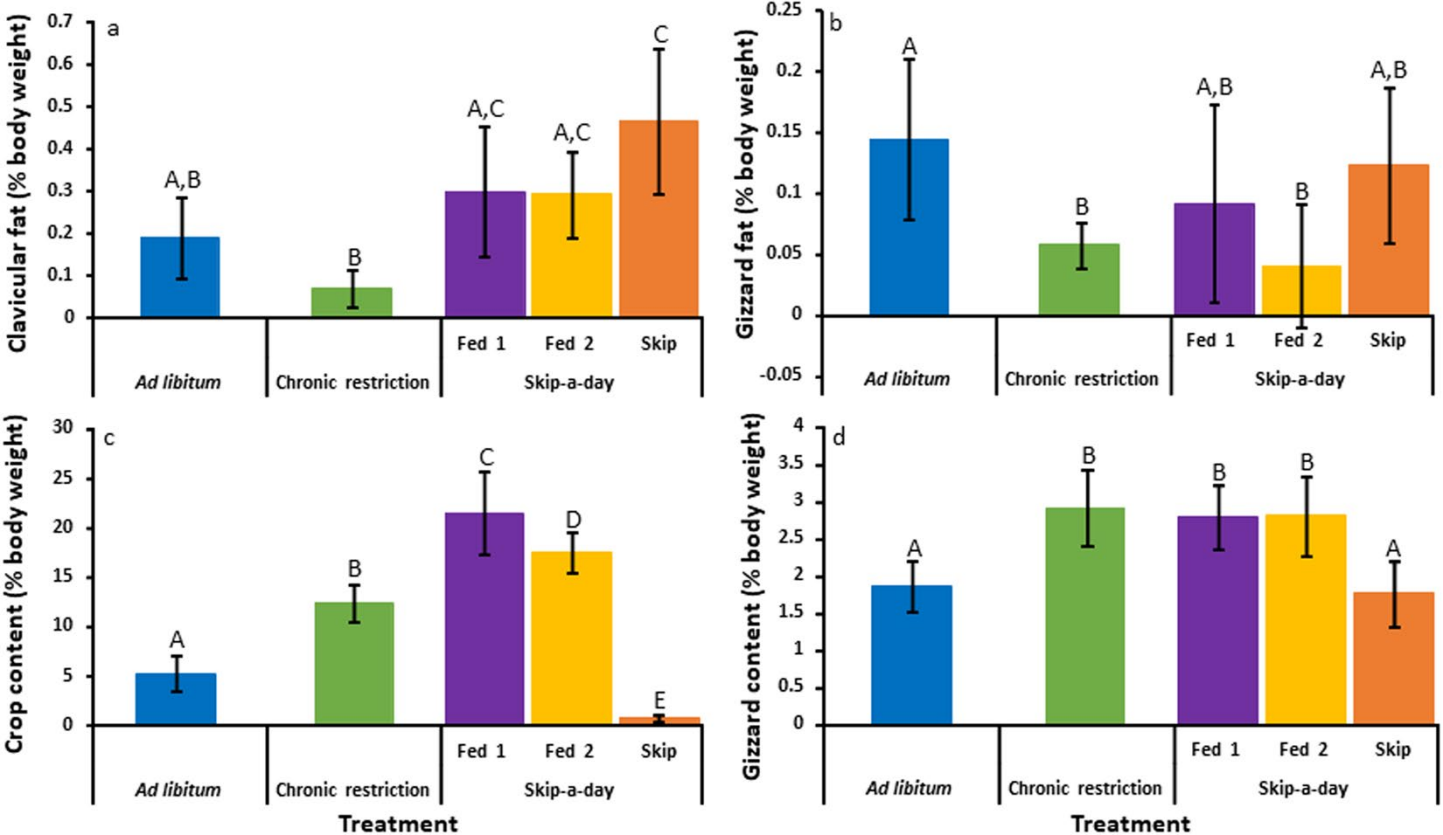
Clavicular fat (**a**), gizzard fat (**b**), crop content (**c**) and gizzard content (**d**) of Red Junglefowl chicks reared under *ad libitum* feeding (blue, N = 6), chronic feed restriction (Green, N = 6) and skip-a-day feeding consisting of a first fed day, Fed 1 (purple, N = 5) a second fed day, Fed 2 (yellow, N = 4) and a skipped day without food (orange, N = 7). Weights are shown as percentages of body weight. Error bars represent standard deviations of the mean. Significant differences (determined using two-way ANOVA, p < 0.05) are indicated with different letters.

**Figure 4:**
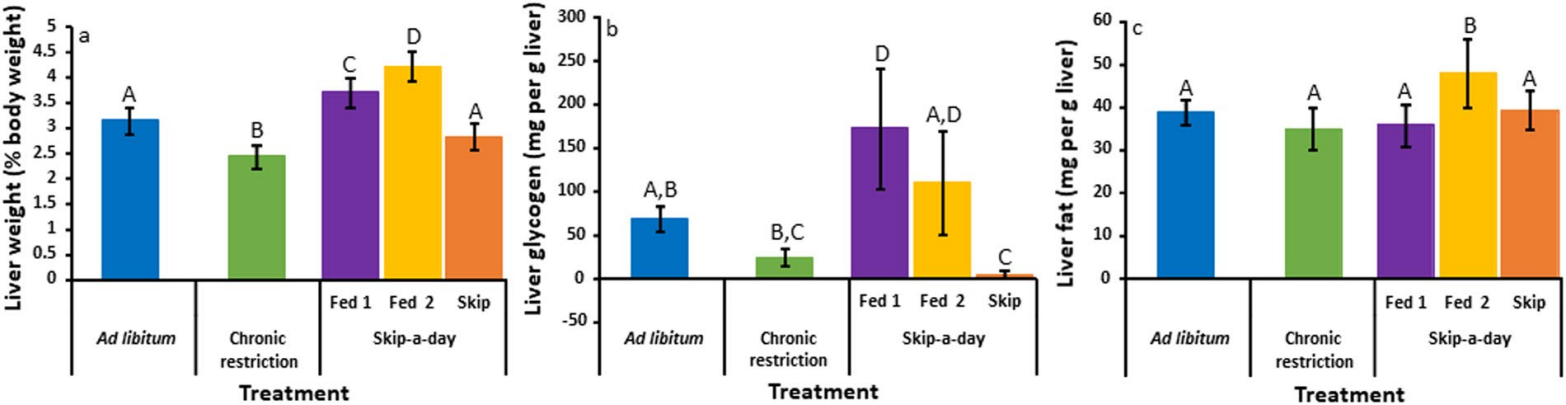
The characteristic weights (**a**), glycogen contents (**b**) and fat contents (**c**) of livers from Red Junglefowl chicks reared under *ad libitum* feeding (blue, N = 6), chronic feed restriction (Green, N = 6) and skip-a-day feeding consisting of a first fed day, Fed 1 (purple, N = 5), a second fed day, Fed 2 (yellow, N = 4) and a skipped day without food (orange, N = 7). Liver weights are shown as a percentage of body weight, liver constituents are shown as mg per g of liver. Error bars represent standard deviations of the mean. Significant differences (determined using two-way ANOVA, p < 0.05) are indicated with different letters.

Clavicular fat (% body mass) was similar between CR and AL birds but was lower in CR individuals in comparison to Fed1, Fed2 and S birds (Fig. 3a, ANOVA: Treatment, F_4,35_ = 10.9, r^2^ = 0.6, p < 0.001). Gizzard fat (% body mass) was more variable across treatments but was lower in CR and F2 birds compared to AL individuals (Fig. 3b, ANOVA: Treatment, F_4,35_ = 4, r^2^ = 0.3, p < 0.01). Crop content (% body mass) was significantly different for all feeding treatments (Fig. 3c, ANOVA: Treatment, F_4,35 _ = 94, r^2^ = 0.9, p < 0.001). Contents were lowest in S birds (mean = 0.82 ± 0.1) and highest in Fed1 birds (mean = 21.4 ± 1.4). Gizzard content (% body mass) was similar between AL and S birds and between CR, Fed1 and Fed2 individuals (Fig. 3d, ANOVA: Treatment, F_4,35_ = 12.3, r^2^ = 0.6, p < 0.001). The weight of the liver (% body mass) differed between feeding groups with the exception of AL and S birds in which liver weights were similar (Fig. 4a, ANOVA: Treatment, F_4,35_ = 54.4, r^2^ = 0.9, p < 0.001). Liver weight was lowest in CR birds and highest in Fed2 birds. Similarly, liver glycogen (mg per g liver) was lower in CR birds in comparison to Fed1 and Fed2 birds but was similar between CR and S treatments (Fig. 4b, ANOVA: Treatment, F_4,34_ = 21.6, r^2^ = 0.6, p < 0.001). Glycogen was lowest in S individuals following 22 hours without access to food. Liver fat (mg per g liver) was less variable and was only significantly elevated in Fed2 birds (Fig. 4c, ANOVA: Treatment, F_4,35_ = 7, r^2^ = 0.4, p < 0.001).

### Linking Carcass traits to gene expression

**Figure 5:**
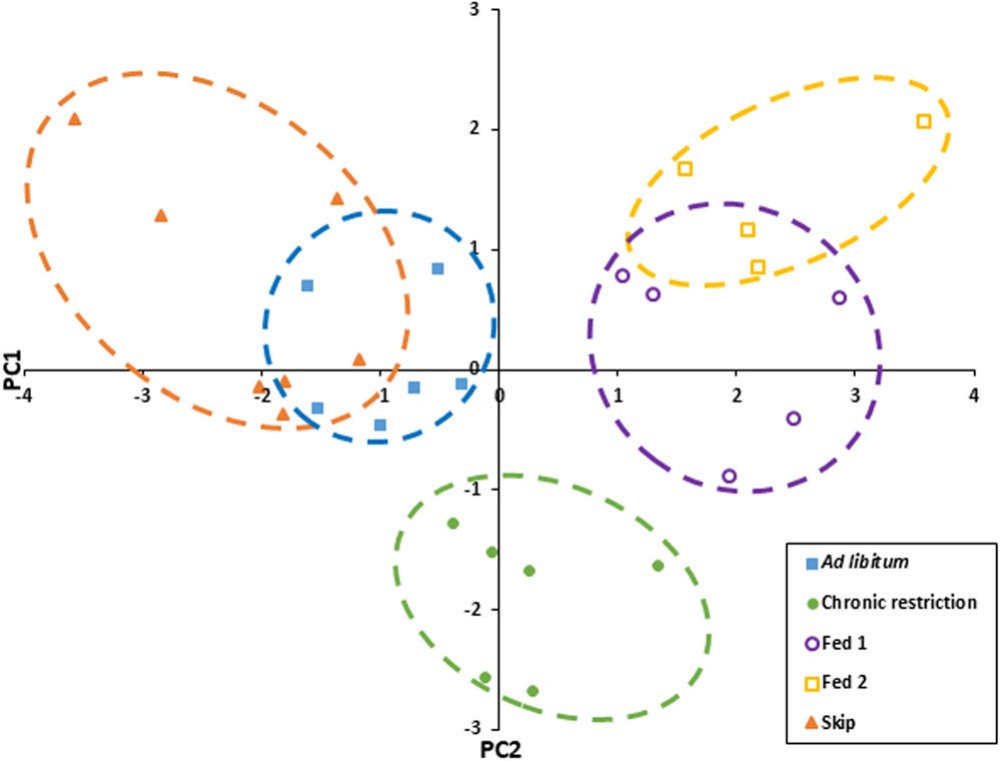
Score plot for the first two principle components representing carcass traits in Red Junglefowl chicks reared under *ad libitum* (blue, filled squares, N = 6), chronic feed restriction (green, filled circles, N = 6) and skip-a-day feeding, consisting of two feeding days, Fed 1 (purple, open circles, N = 5) and Fed 2 (yellow, open squares, N = 4), followed by a skip day (orange, filled triangles, N = 7).

**Figure 6:**
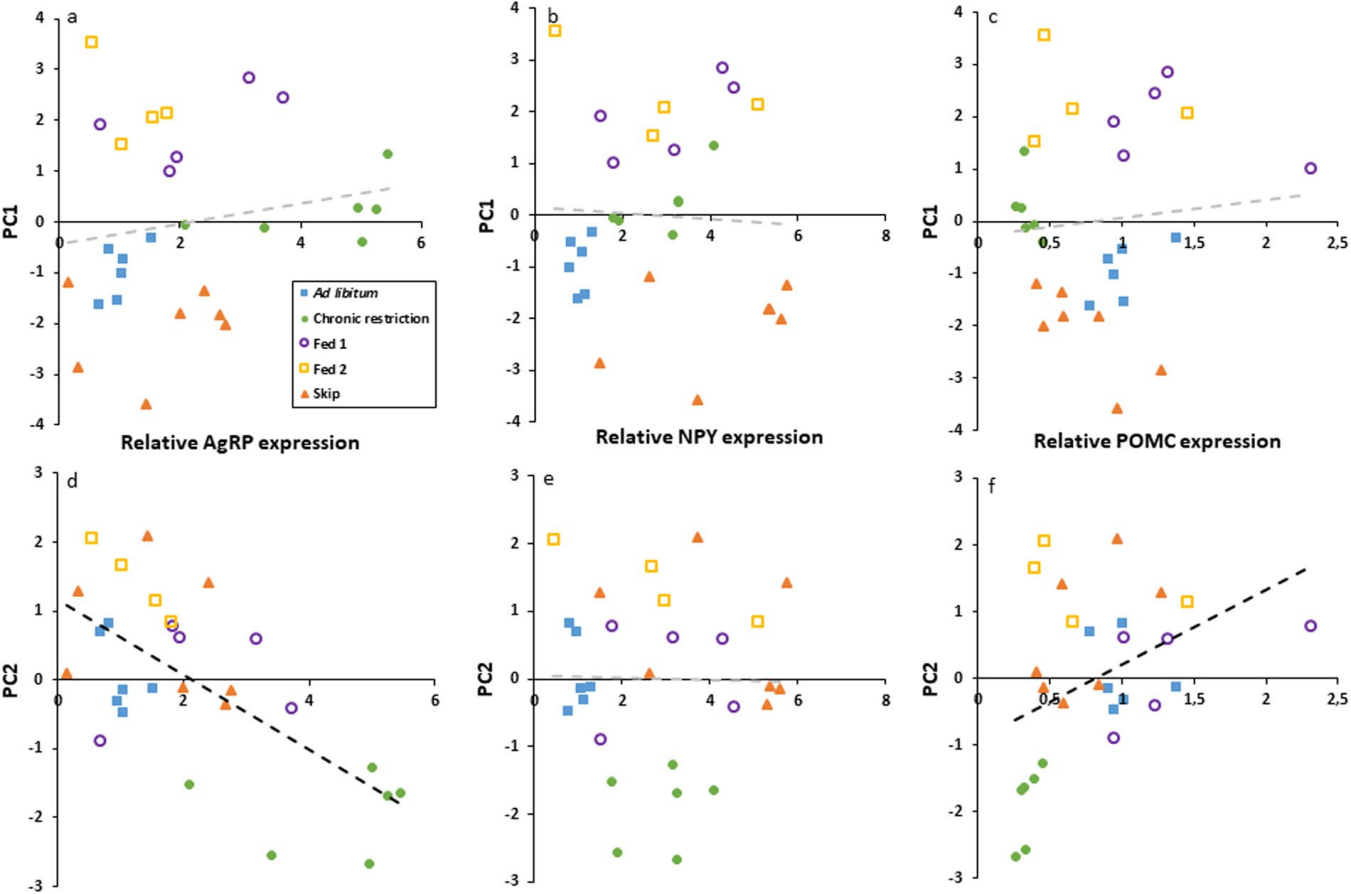
Individual regressions of the first two principal components (representing the variability in measured carcass traits) with basal hypothalamic gene expression of agouti-related peptide (AgRP, a/d), neuropeptide Y (NPY, b/e) and pro-opiomelanocortin (POMC, c/f) for Red Junglefowl chicks reared under *ad libitum* (blue, filled squares, N = 6), chronic feed restriction (green, filled circles, N = 6) and skip-a-day feeding, consisting of two feeding days, Fed 1 (purple, open circles, N = 5) and Fed 2 (yellow, open squares, N = 4), followed by a skip day (orange, filled triangles, N = 7). Significant associations between variables (regression analysis, p < 0.05) are marked in bold, non-significant associations are shown in grey.

Clavicular fat, gizzard fill, gizzard fat and liver mass showed significant associations with one or more of the hypothalamic genes (Table 1). AgRP was positively associated with gizzard fill (F_1,27_ = 7.5, r^2^ = 0.22 p < 0.05) and negatively associated with gizzard fat (F_1,27_ = 6.2, r^2^ = 0.19 p < 0.05) and liver mass (F_1,27_ = 6.1, r^2^ = 0.19 p < 0.05). Conversely POMC showed a positive association with % liver mass (F_1,27_ = 7.9, r^2^ = 0.23 p < 0.01). NPY was not associated with any of the carcass traits measured. Condensing the data using PCA, the first two principle components represented 70% of the variation in the measured carcass traits (Fig. 5, supplementary table 1). PC1 was strongly correlated with factors related to recently ingested food, such as increased crop fill (loading = 0.5) and gizzard fill (loading = 0.42) and also with liver glycogen (loading = 0.43) and decreased gizzard fat (loading = −0.41). Alternatively, PC2 was correlated with fat indicators such as increased clavicular (loading = 0.52) and liver fat (loading = 0.53). Regression analysis showed PC1 to not be significantly associated with any of the hypothalamic genes measured, however PC2 showed a significant negative and positive relationship with AgRP (F_1,27_ = 19.6, r^2^ = 0.43, P < 0.001) and POMC (F_1,27_ = 5.2, r^2^ = 0.17, p < 0.05), respectively (Fig. 6).

**Table 1:** The results of individual regression analyses of carcass traits with relative basal hypothalamic gene expression levels.

Carcass trait	AgRP				NPY				POMC			
	N	r^2^	F	P	N	r^2^	F	P	N	r^2^	F	P
Clavicular fat (%)	**28**	**0.14**	(**1,27**)** = 4.27**	**0.049**	28	0.1	(1,27) = 2.72	0.11	28	0.04	(1,27) = 0.96	0.34
Crop fill (%)	28	0.04	(1,27) = 1.19	0.29	28	0.002	(1,27) = 0.06	0.81	28	0.002	(1,27) = 0.06	0.81
Gizzard fill (%)	**28**	**0.22**	(**1,27**)** = 7.47**	**0.01**	28	0.003	(1,27) = 0.08	0.78	28	0.008	(1,27) = 0.2	0.66
Gizzard fat (%)	**28**	**0.19**	(**1,27**)** = 6.19**	**0.02**	28	0.09	(1,27) = 2.72	0.11	28	0.05	(1,27) = 1.45	0.24
Liver mass (%)	**28**	**0.19**	(**1,27**)** = 6.12**	**0.02**	28	0.006	(1,27) = 0.15	0.71	**28**	**0.23**	(**1,27**)** = 7.92**	**0.009**
Glycogen (mg per g liver)	28	0.02	(1,27) = 0.53	0.47	28	0.06	(1,27) = 1.64	0.21	28	0.1	(1,27) = 2.98	0.1
Fat (mg per g liver)	28	0.08	(1,27) = 2.22	0.15	28	0.002	(1,27) = 0.04	0.85	28	0.005	(1,27) = 0.14	0.71

Significant associations are shown in bold.

## Discussion

The three different feeding regimens utilized for this experiment elicited clear differences in the pattern of food intake, growth and physiology. As predicted, CR birds grew more slowly than AL birds but were not significantly different in mass from the skip-a-day birds despite consuming less food over a three-day period. Similar growth depressions in response to intermittent feeding have been observed in farmed chickens^21^. Skip-a-day birds show fluctuations in their levels of adipose tissue and liver glycogen as a result of intermittent feeding. The lower feed conversion efficiency in comparison to CR birds may therefore lie in the energy losses associated with the acquisition and utilisation of peripheral fat stores^21,22^.

The level of crop fill 4 hours after feeding differed in all treatments. CR birds had nearly three times the crop fill of AL birds despite being fed less than AL individuals, indicating that birds were hyperphagic in order to achieve their desired energetic status. The level of this ‘binge eating’ is likely to be indicative of the hunger status of birds at the time of feeding and was therefore highest in Fed1 skip-a-day birds following 42 hours of feed restriction and was reduced the following day (Fed2). The unpredictability of food brought about by restrictive meals may shift the optimal feeding strategy towards securing large amounts of feed rapidly despite the increased costs of carrying food equivalent to 20% of body weight. Such extreme crop filling seen in skip-a-day birds illustrates the flexibility of the crop as a cache for food acquisition and storage when food availability is restricted. Given that both skip-a-day and CR birds had significantly heavier crops than AL birds (supplementary figure 1a), the process of binge eating itself appears to result in permanent morphological changes. Such anatomical changes in response to intermittent feeding have previously been observed in domestic chickens trained to eat their daily food during a 2 hour period^23^. Similarly, the gizzards of CR birds were relatively heavier than those of AL individuals (supplementary figure 1b). Although the gizzard’s primary function is in the breakdown of ingested food, gizzard contents were also elevated in both CR and skip-a-day birds after feeding, albeit at a reduced level compared to the crop. These differences in gizzard contents may be the result of differences in gizzard size or alternatively indicative of differences in the volume of feed moving through the gut.

The experimental feeding regimens had strikingly different effects upon the weight of the liver, associated with changes in glycogen and fat content. CR had the lowest relative liver masses despite no significant differences in liver glycogen or fat compared to AL birds. Skip-a-day birds on the other hand showed large fluctuations in liver mass through their 3-day feeding cycle. These large changes in liver weight have been seen previously in broiler breeder chickens and are indicative of large swings in nutritional status^24^. In broiler breeders, changes in liver mass during skip-a-day feeding are associated with profound increases in liver fat and glycogen content 24 hours after refeeding following a period of fast. Similarly, leghorn chickens fed meals for 2 hours per day also show a postprandial doubling of liver glycogen^25^. Here, glycogen in the liver shows a large and rapid increased just 4 hours after refeeding following 42 hours of feed restriction, which returns towards AL levels after a second day of feeding. The rise in liver fat is less rapid but, as in broiler chickens, peaks 24 hours after refeeding^24^. It appears, therefore, that domestication has had little impact upon the metabolic response of the liver. It would be of future interest to determine if these similarities in gross morphological changes are mirrored in the hepatic lipid metabolism of domestic and wild chickens.

Taken together, the physiological measures and carcass traits suggest that, despite slowed growth, skip-a-day birds are able to adapt to the feeding regimen and the associated 42 hour fasting period. Hyperphagia on fed days compared to CR birds facilitates the acquisition of elevated liver glycogen and adipose stores which fulfil the energy requirements of fasting days. CR birds, on the other hand, are chronically unable to acquire the energy surplus needed to build energy reserves despite demonstrating hyperphagia.

Previous studies suggest that AgRP expression within the arcuate nucleus provides an integrated measure of food intake history, responding to both the long-term and immediate feeding state of birds. Hypothalamic AgRP mRNA increases in both acutely and chronically fasted individuals^5,7^ however, upon refeeding, the time required to reach *ad libitum* levels appears to be proportional to the duration of prior restriction. AgRP may therefore be an indicator of a bird’s deviation from a desired physiological setpoint, most likely in terms of peripheral energy stores. Here, the measured AgRP expression levels represent those 4 hours after the delivery of new feed, with the exception of S birds which have received no food. In this scenario, Fed1 birds were refed after a fasting period of 42 hours. Despite these vastly different feeding states, hypothalamic AgRP is only elevated above AL levels in CR individuals, supporting the notion that AgRP represents long-term feeding history. The finding that AgRP is not elevated in S birds is, however surprising as these individuals are both chronically (based upon their reduced cumulative feed intake (Fig. 1c)) and acutely restricted in comparison to AL birds. The reason for the unresponsive AgRP signal in S birds may lie in their elevated adiposity. The acquisition of fat on fed days may be sufficient to fuel the metabolic requirements of the birds during periods of fasting, thereby reducing their energy deficit (both chronic and short-term) in comparison to CR individuals^21^. Boswell and Dunn (2017) suggested that AgRP may represent a measure of growth potential in broiler breeder chickens, with higher expression levels indicating larger deviations from the natural growth trajectory. Here, differential expression of AgRP between restricted and skipped birds in the absence of size differences indicate that this is not the case in red junglefowl.

Although NPY responds to both chronic and acute feed restriction in a manner similar to that of AgRP in birds^11,12^, Dunn *et al*.^5^, suggested that NPY is less discriminatory to the feeding status in broilers. Indeed, broiler chicks fasted for 24 hours showed no hypothalamic NPY response^7^. Here, NPY was elevated to a small amount above AL levels under all feeding regimens, suggesting a level of responsiveness to chronic restriction. However, only S birds showed a statistically significant elevation in NPY above that of AL birds. These data indicate an acute temporal responsiveness of NPY to the immediate lack of food experienced by S birds that is absent in the AgRP signal.

The role of POMC in driving avian hunger is currently unclear as a result of inconsistent findings regarding its response to feed restriction. Here, the relative changes in POMC resulting from the CR and S feeding regimens were small in comparison to those of AgRP and NPY. Chronic restriction did, however, significantly diminish the presence of POMC mRNA even after 4 hours of refeeding, indicating a response to chronic feed restriction. Such a chronic response was also seen in 3 week old broiler and layer chicks^26^ but not in 12 week old broiler breeders^5^. Acute restriction, on the other hand, appears to have little effect on the POMC response.

The differences in gene expression presented are primarily the result of differential expression within the arcuate nucleus of the basal hypothalamus. The arcuate nucleus acts as an intermediary, integrating both humoral, peripheral indicators of energy status with circadian and sensory information regarding the time of day and presence of food, respectively^27,28^. In turn, both intra- and extra-hypothalamic projections from the arcuate nucleus elicit behavioural and metabolic responses which summate as an overall sensation of hunger or satiety. The present gene expression data illustrate that distinct neuronal populations, primarily within the arcuate nucleus exhibit characteristic neuroendocrine responses dependent on the temporal delivery of feed restriction. How these expressional changes are subsequently translated into the perception of hunger in birds is currently unknown. Here, the elevated AgRP and decreased POMC expression in CR birds after feeding suggests that these individuals are experiencing a chronic feeling of hunger. However, if assessed by the same criteria, S birds appear not to be experiencing chronic hunger despite fasting every three days. Hypothalamic gene expression alone may therefore not be a reliable index of hunger. One potential discrepancy lies in the fact that here, only mRNA levels were measured and not absolute values of the coded neuropeptides. Changes in avian hypothalamic AGRP- immunoreactive cell numbers in response to nutritional status, however suggest that mRNA levels are indicative of the downstream protein response^29^. In order to further understand how the transcriptional state of the arcuate nucleus translates into hunger in a context-dependent manner, future work should focus on integrating both feed-intake, gene expression, peptide levels and behavioural indices of hunger.

The basal hypothalamus, in particular the arcuate nucleus is regarded as one of the key sites for the integration of peripheral hunger and satiety signals. Any association (or lack thereof) between external physiological measures and the hypothalamic expression of ‘hunger genes’ will therefore provide an insight into the peripheral signals relevant to hypothalamic hunger and the central effectors that they inform. However, when looking for associations between individual carcass traits and gene expression, few of the individual regressions showed significant associations. Strong candidate signals were those indicating the amount of recently ingested food (crop and gizzard content) and those indicating longer term energy stores (liver glycogen, liver fat and clavicular fat). Despite being a direct indicator of food ingestion, crop fill was not associated with any of AgRP, POMC or NPY expression. Indeed, birds with dramatic differences in crop fill such as Fed2 birds and S birds showed no differences in any of the hypothalamic genes. Gizzard fill was, however, strongly associated with AgRP expression as was the mass of fat around the gizzard. Clavicular fat, perhaps the most robust indicator of stored energy was only weakly associated with AgRP (p = 0.049) and the glycogen and fat in the liver were similarly poor in predicting the level of any of the genes measured.

Given the multiple physiological and behavioural responses of the birds to different feeding regimens and the integrative nature of the arcuate nucleus response, it is perhaps unsurprising that single measures alone do not show strong associations with hypothalamic ‘hunger genes’.

Instead, the data may be best described using principal component analysis in which the overall phenotypic responses of birds are grouped and subsequently correlated with gene expression. By performing such an analysis, individuals clustered according to the first two principal components, which explained around 70% of the variance. The first principle component was associated with feed in the digestive tract (the level of crop and gizzard fill) as well as gizzard fat and liver glycogen, whereas the second principal component was associated with stored fat (clavicular fat and liver fat). Interestingly, none of the variance in the first principle component was explained by the expression of AgRP, NPY or POMC, suggesting that there is no hypothalamic satiety signal associated with the volume of food in the upper digestive system. This disconnect between the crop fill and hypothalamic satiety may explain previous findings from artificial crop and gizzard filling experiments in which artificially filling the crop of fasted turkeys prior to meal initiation does not influence subsequent meal size^30^. The severe feed restriction of the parental birds of broiler chickens in the poultry industry is a global welfare issue. It has been suggested that the dilution of feed with low energy bulking materials (qualitative restriction) could reduce the hunger associated with such quantitative restriction. In agreement with previous studies assessing the welfare of qualitatively restricted birds, our data suggest that increasing crop fill does not necessarily increase satiety^31,32^.

Principle component two, representing the level of stored energy was strongly associated with AgRP and to a lesser extent with POMC but not with NPY. Dunn, 2015 suggested that AgRP level was related to the level of peripheral energy stores. It is well established in mammals, that the adipose-derived peptide, leptin, is the primary indicator of peripherally stored energy in the form of fat and therefore inhibits feeding, in part through the inhibition of AgRP. In birds, the role of leptin appears to be diminished, with low circulating levels and mixed results from experiments involving peripheral administration of the hormone^19^. The data here demonstrate that AgRP expression is responsive to peripheral fat stores, decreasing with fat indices as observed in rodents. This association has not, to our knowledge, been demonstrated previously in birds. The observation that NPY showed no association with either principal component 1 or 2 suggests that its stimulatory signal is not one of those measured and may be separate from that of AgRP. Mean values of NPY were highest (although not statistically significantly) in birds undergoing a skip day without food. NPY therefore appears to be diminished as a result of more immediate signals of food intake and satiation as opposed to longer term indicators of energy status. These are likely to include post-ingestive gastrointestinal peptides such as cholecystokinin as well as post-absorptive circulating products of recent digestion such as glucose (and insulin), amino acids and free fatty acids.

One challenge in determining the relationship between indices of energy status (such as adipose tissue) and the hypothalamic expression of ‘hunger genes’ is that often, feed restriction experiments decrease these indices in parallel with the level of restriction. For example, when the level of feed is high, body fat will be high and vice versa. Here, the skip-a-day regimen provides novel insights into the signalling response of the hypothalamus to different hunger scenarios as the acquisition of fat (a signal of long term energy status) peaks at the same time that there is an absence of food in the short term (i.e. on Skip days). In these different scenarios, both S and CR birds appear to be hungry, albeit in two distinct ways. CR birds are hungry in the chronic sense as they have no external stores of fat and therefore show high AgRP and low POMC levels. They have, however, been feeding and so are more satiated in the immediate sense, reflected in the unchanged levels of NPY. S birds, conversely are less hungry in the chronic sense as they have peripheral adipose stores (and therefore AL levels of AgRP) but have not eaten in the immediate sense and so are hungry according to NPY. These findings may be of relevance to the broiler farming industry in which broiler breeders are feed restricted either by quantitative restriction or skip-a-day feeding^33^. It is not clear from the data presented here whether perpetual, chronic hunger is ethically more suitable than intermittent, immediate hunger but certainly future work should address this question.

In summary, the present data provide novel insights into the response of ‘hunger genes’ within the basal hypothalamus to differing gradations of hunger and to peripheral indices of energy availability. In particular, quantitative and temporal feed restrictions elicit different patterns of fat storage that are reflected in AgRP and POMC, but not NPY expression. These data provide new insights into avian hypothalamic hunger beyond those from chronic feed restriction experiments but reinforce the notion that AgRP and NPY perform distinct functional roles in contributing to the perception of hunger.

## Methods

### Animals

Red Junglefowl were raised indoors in pens (260 × 120 cm). The population originated from a wild population in Thailand (see Schütz *et al*.^34^ for details about the origin of used populations in the experiment). Birds were initially fed *ad libitum* until day 14, at which point they were switched to one of three feeding regimens. These consisted of the following:

1. Group 1) *Ad libitum* feeding (AL, N = 48)
2. Group 2) Chronic feed restriction (CR, N = 48) in which birds were fed 60% (days 14–24) and 75% (day 25 onwards) of the age-matched AL food intake.
3. Groups 3 and 4) skip-a-day feeding (N = 43 and 40, groups 3 and 4, respectively) in which birds were fed 150% of their age-matched AL intake on two consecutive days (referred to as Fed1 and Fed2), followed by a third day in which food was skipped (referred to as S).

Water was provided *ad libitum*. Throughout the experiments, feed consisted of a grower pellet (Penna, Lantmännen Lantbruk, Malmö) consisting of 18.4% crude protein and 2.8% crude fat. Temperatures were maintained at 26.8 ± 0.6 °C (AL and CR) and 28.2 ± 1 °C (skip-a-day) and the photoperiod was 12 L:12D. Mean humidity was 30.6 ± 1.1% (AL and CR birds) and 19.5 ± 0.6% (skip-a-day birds).

In order to dissociate the presence of investigators from the delivery of food, birds were fed by means of a motorized partition. Whilst in the ‘open’ position, the feeding area was accessible and whilst ‘closed’ the feeding area was inaccessible. AL and CR birds had access to the feeding area throughout the day with the exception of a 20 minute period at midday (zeitgeber time = 12:00) during which the area was closed. At this time, uneaten food from the preceding 24 hours was removed for weighing and food for the next 24 hours was added. For skip-a-day groups, food was only accessible for 6 hours starting from midday. At the end of this 6 hour period, food was removed for weighing and the ration for the following day was added. Upon removal from the pen, food was processed by sieving and sedimentation to remove faecal matter and sawdust. The processed food was then dried and weighed to determine food intake.

All experimental protocols were approved by Linköping Council for Ethical Licensing of Animal Experiments, ethical permit ID638. Experiments were conducted in accordance with the approved guidelines.

### Dissections

At the end of the experimental period, between 16:00 and 17:00, birds were killed by decapitation. AL and CR birds were sampled at 36 days of age and skip-a-day birds were sampled at between 36 and 42 days of age. Livers were immediately excised, weighed and frozen in liquid nitrogen. Brains were immediately excised and the basal hypothalamus was removed and frozen in liquid nitrogen. Dissections extended laterally 1 mm either side of the third ventricle and dorsally 2 mm from the ventral surface of the median eminence as far as the intrahypothalamic border as in Dunn *et al*.^5^. This region contains the arcuate nucleus which was of primary interest, as well as the ventromedial hypothalamus and lateral tubular nucleus. Carcass data were also recorded consisting of the mass of fat tissue surrounding the clavicles and gizzard, as well as the weight of the gizzard and crop contents. All samples were stored at −80 °C prior to analysis.

### Liver glycogen and lipid content

Lipids were extracted from liver samples according to the method of Folch *et al*.^35^. Approximately 250 mg of tissue was homogenized in chloroform and methanol solution using a dispersing instrument (T10 basic ultra-turrax®, IKA, Staufen). After mixing (2 hours at 4 °C), 1 ml of water with 0.02% CaCl was added to the homogenate before centrifugation (20 minutes at 1800 rpm). The upper phase of the mixture was discarded and the interface was rinsed three times with 1 ml pure solvents upper phase (chloroform, methanol and water in the ratio 3:48:47). The solution was then filtered through fat-free filter paper and air dried at 60 °C for 24 hours to evaporate the chloroform. The remaining lipid residue was then weighed.

Liver glycogen was extracted according to the method of Maatjens *et al*.^36^. Briefly, around 300 mg of tissue was homogenized in 7% HClO_4_ using a dispersing instrument (T10 basic ultra-turrax®, IKA, Germany). The resultant suspension was centrifuged (15 minutes at 2900 x g and 4 °C), the supernatant removed and washed with 1 mL of petroleum ether. Samples were frozen at −80 °C prior to analysis. Glycogen was determined using an iodine binding assay^37^ and hepatic bovine glycogen (type IX, Sigma-Aldrich, Germany) was used as a standard.

### Gene expression

AgRP, POMC and NPY mRNA in the basal hypothalamus was quantified using quantitative polymerase chain reaction (qPCR). Total, phenol-free RNA was first isolated from tissue using the RNAqueous®-Micro kit (Thermo Fisher Scientific, USA) and reverse transcribed for qPCR. Gene-specific primers amplifying AgRP (forward primer: 5’-GGAACCGCAGGCATTGTC-3′, reverse primer: 5′-GTAGCAGAAGGCGTTGAAGAA-3′), POMC (forward primer: 5′-CGCTACGGCGGCTTCA-3′, reverse primer: 5′-TCTTGTAGGCGCTTTTGACGAT-3′) and NPY (forward primer: 5′-GAGGCACTACATCAACCTCATCAC-3′, reverse primer: 5′-TGTTTTCTGTGCTTTCCCTCAA-3′) were taken from Lei and Lixian^7^. Real-time PCR was run on a LightCycler® 480 instrument II (Roche Molecular Systems, USA) with the conditions: 95 °C for 4 minutes, 40 cycles of 95 °C for 30 s, 60 °C for 30 s and 72 °C for 10 s. Reactions were run using 1 µl of cDNA, 5 µl of SYBR green mastermix and 0.5 µl of primers (500 nM).

Relative quantification of expression was calculated using equation 1 (taken from^38^).

1 $${\rm{ratio}}=(\frac{{({E}_{{\rm{ref}}})}^{{{\rm{CP}}}_{{\rm{sample}}}}}{{({E}_{{\rm{target}}})}^{{{\rm{CP}}}_{{\rm{sample}}}}}\,)\div\,\bar{x}(\frac{{({E}_{{\rm{ref}}})}^{{{\rm{CP}}}_{{\rm{calibrator}}}}}{{({E}_{{\rm{target}}})}^{{{\rm{CP}}}_{{\rm{calibrator}}}}})$$

where *E*_ref_ is the efficiency of the housekeeper reference gene, GAPDH (forward primer: 5′-GTCAAGGCTGAGAACGGGAA-3′, reverse primer: 5′-GCCCATTTGATGTTGCTGGG-3′), *E*_target_ is the efficiency of the target gene and CP_Sample_ and CP_Calibrator_ are the crossing points of the sample of interest and the reference samples used for calibration. Here, data for AL birds were used as the calibrator values.

### Statistics

Two-way ANOVA was used to test for differences in gene expression and carcass traits between the treatment groups. Prior to statistical analysis, data were tested for equality of variances. Sex was included in all models but was only found to influence body mass. Where the interaction term (treatment*sex) was found to be insignificant (p > 0.05), the model was simplified and re-run omitting the interaction term. Tukey post hoc test were used to test for differences in the main effects. Linear regressions were used to test for association between gene expression and carcass traits (after establishing normality using a Ryan Joiner test). In order to condense the carcass trait data into fewer latent variables, a principal component analysis was performed and eigenvalues were obtained from the correlation matrix. Regression analysis was then used to establish any associations between the first two axes (representing 70% of the variation) and gene expression levels. All data presented as mean ± standard error of the mean unless otherwise stated. All carcass data are presented as percentages of lean body mass (body mass with the fill of the crop subtracted), with the exception of liver glycogen and fat which are presented as mg per g liver. All statistical analyses were performed in Minitab version 18.1 (Pennsylvania, USA).

## Electronic supplementary material

Supplementary information
