# Supplementary material for: The physiological and neuroendocrine correlates of hunger in the Red Junglefowl (Gallus gallus)
Source: Sci Rep. 2017 Dec 21;7:17984. doi: 10.1038/s41598-017-17922-w (PMC5740172; doi:10.1038/s41598-017-17922-w)
Supplement: Supplementary file 1 — Supplementary information [file 41598_2017_17922_MOESM1_ESM.pdf]

Supplementary information:

The physiological and neuroendocrine correlates of hunger in the Red Junglefowl (*Gallus gallus*)

*Lees, J.J.[1], Lindholm, C. [1] , Batakis, P. [1] , Busscher, M.[2] and Altimiras, J. [1]

[1] IFM, University of Linköping, Linköping, Sweden

[2] Department of Animal Sciences, Wageningen University, Wageningen, Netherlands

*Correspondence to: John Lees, IFM, University of Linköping, Linköping, 58183, Sweden

(john.lees@liu.se)

Variable	PC1	PC2	PC3
Clavicular fat (%)	-0.25	0.52	-0.25
Crop fill (%)	0.50	-0.08	-0.24
Gizzard fill (%)	0.42	-0.32	-0.13
Gizzard fat (%)	-0.41	0.17	-0.49
Liver mass (%)	0.35	0.49	-0.29
Glycogen (per g liver)	0.43	0.26	-0.25
Fat (per g liver)	0.18	0.53	0.69

	PC1	PC2	PC3
Eigenvalue	3.29	1.64	0.76
Proportion	0.47	0.24	0.11
Cumulative	0.47	0.70	0.81

Supplementary table 1: Principal component analysis of Red Junglefowl chick carcass traits in response to different feeding regimens.

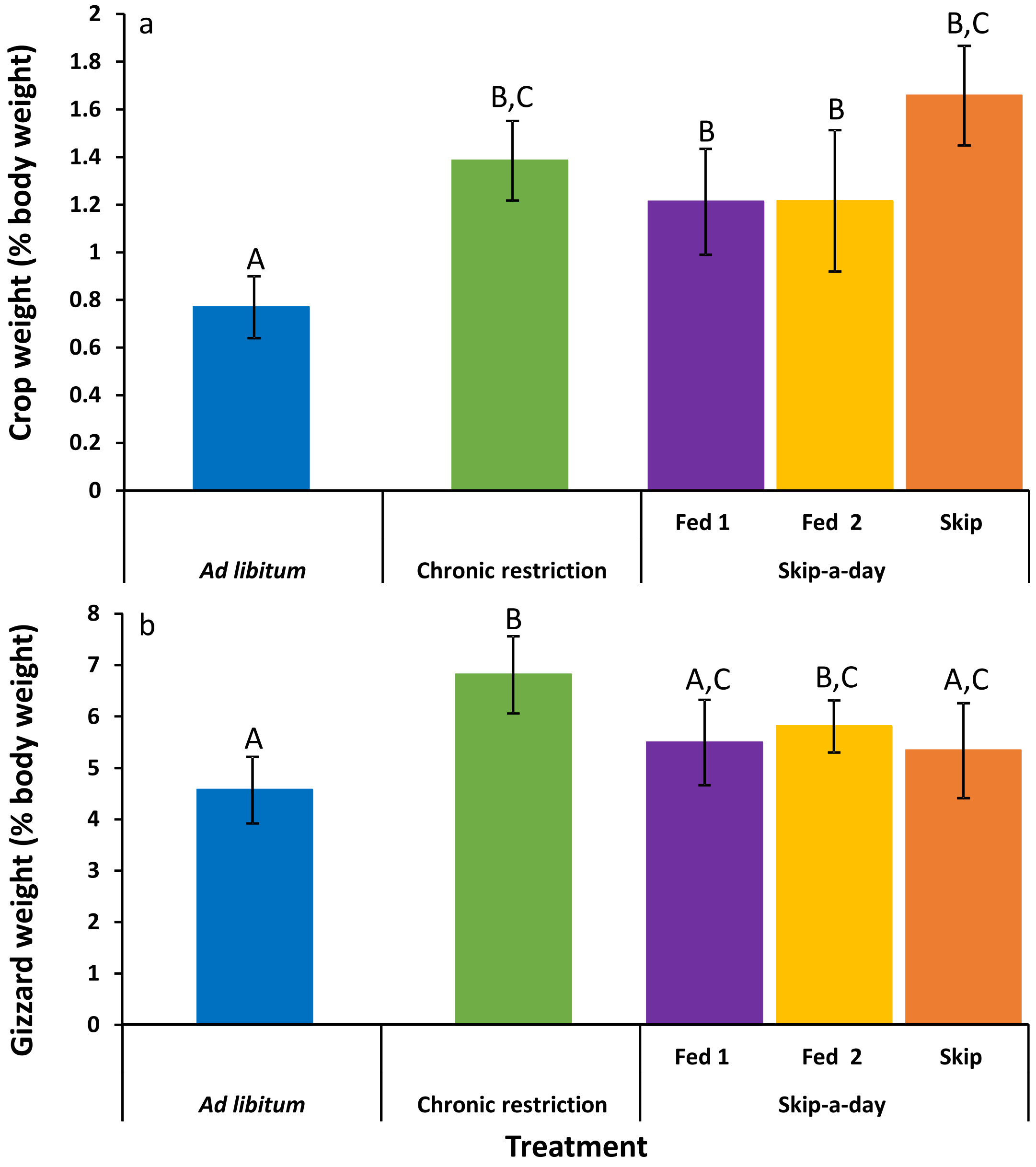

Supplementary figure 1: Crop and gizzard weights of Red Junglefowl chicks reared under different feeding regimens. Weights are shown as a percentage of body weight.
